# The impact of histologic subtype on primary site surgery in the management of metastatic lobular versus ductal breast cancer: a population based study from the National Cancer Database

**DOI:** 10.21203/rs.3.rs-3137103/v1

**Published:** 2023-07-19

**Authors:** Harriet T Rothschild, Elle N Clelland, Mary Kathryn Abel, A Jo Chien, Amy M Shui, Laura Esserman, Seema A Khan, Rita A Mukhtar

**Affiliations:** University of California San Francisco School of Medicine; University of California San Francisco School of Medicine; University of California San Francisco School of Medicine; University of California San Francisco Department of Medicine; University of California San Francisco Department of Epidemiology and Biostatistics; University of California San Francisco Department of Surgery; Northwestern University Department of Surgery; University of California San Francisco Department of Surgery

**Keywords:** Lobular, NCDB, Metastatic, Surgery

## Abstract

**Purpose:**

Primary site surgery for metastatic breast cancer improves local control but does not impact overall survival. Whether histologic subtype influences patient selection for surgery is unknown. Given differences in surgical management between early-stage lobular versus ductal disease, we evaluated the impact of histology on primary site surgery in patients with metastatic breast cancer.

**Methods:**

The National Cancer Database (NCDB, 2010–2016) was queried for patients with stage IV HR-positive, HER2-negative invasive lobular carcinoma (ILC) and invasive ductal carcinoma (IDC). We compared clinicopathologic features, primary site surgery rates, and outcomes by histologic subtype. Multivariable Cox proportional hazard models with and without propensity score matching were used for overall survival (OS) analyses.

**Results:**

In 25,294 patients, primary site surgery was slightly but significantly less common in the 6,123 patients with ILC compared to the 19,171 patients with IDC (26.9% versus 28.8%, *p* = 0.004). Those with ILC were less likely to receive chemotherapy (41.3% versus 47.4%, *p* < 0.0001) or radiotherapy (29.1% versus 37.9%, *p* < 0.0001), and had shorter OS. While mastectomy rates were similar, those with ILC had more positive margins (10.6% versus 8.3%, *p* = 0.005). In both groups, the odds of undergoing surgery decreased over time, and were higher in younger patients with T2/T3 tumors and higher nodal burden.

**Conclusion:**

Lobular histology is associated with less primary site surgery, higher positive margin rates, less radiotherapy and chemotherapy, and shorter OS compared to those with HR-positive HER2-negative IDC. These findings support the need for ILC-specific data and treatment approaches in the setting of metastatic disease.

## Introduction

Invasive lobular carcinoma (ILC) is the second most common histological subtype of breast cancer after invasive ductal carcinoma (IDC), representing 10–15% of all cases [1]. Many studies have demonstrated differences between ILC and IDC, in both early and advanced stages [2]. In the metastatic setting, ILC differs in its pattern of metastatic sites, often involving the bone, and gastrointestinal tract [2, 3, 4]. Additionally, several studies demonstrate worse overall survival (OS) in metastatic ILC compared to IDC, even when evaluating patients with similar receptor subtypes [4, 5, 6].

While investigators have evaluated surgical outcomes by histologic subtype in early-stage disease, there are scant data evaluating the use of primary site surgery in the metastatic setting in those with ILC versus IDC. The current recommended therapy for metastatic breast cancer is systemic therapy, with local therapy reserved for palliation of symptoms [7]. While retrospective studies and institutional series have found associations between primary tumor resection and longer survival in those with metastatic breast cancer [8], most randomized control trials have not demonstrated such a survival advantage [9, 10, 11]. A previous study found that ILC patients with bone-only metastases had longer OS than those with metastases to multiple organs when given a combination of chemotherapy and surgery, but it is unclear whether this reflects the more indolent course of osseous metastases, or an ILC specific effect of treatment [4, 12].

As such, whether histologic subtype should factor into patient selection for primary site surgery is unknown. Prior analyses and a prospective registry have suggested that if surgery of the primary tumor is associated with improved survival, this may be more likely in those with hormone receptor (HR) positive disease, or bone only metastases [10, 13]. Given that ILC is known to be largely HR-positive, HER2-negative, and has a propensity for bone metastases, we wondered if practices in surgical resection of the primary would differ or be associated with differential outcomes in patients with metastatic ILC compared to IDC.

In this study we used the National Cancer Database (NCDB) to evaluate differences in practice patterns and management of patients with metastatic ILC compared to metastatic IDC. Specifically, we investigated the following questions: whether the rates of primary site surgery differ by histologic subtype and whether the selection factors associated with undergoing primary site surgery differ by histologic subtype. As secondary endpoints, we evaluated the use of chemotherapy and radiotherapy relative to surgery by histologic subtype, and the association between primary site surgery and OS in ILC and IDC cohorts in both unmatched and propensity score matched multivariable models.

## Methods

### Data Source and Study Cohort

The NCDB is a national comprehensive clinical surveillance resource maintained by the American College of Surgeons and the American Cancer Society. It represents over 70% of all newly diagnosed cancer cases in the United States and includes patient demographics, clinical information, and survival outcomes [14, 15]. For the analysis, Participants User Files (PUF) from 2010–2016 were used. Due to the de-identified nature of the public-access user files, the study was deemed exempt from institutional review board approval.

Since most ILC tumors are HR-positive and human epidermal growth factor receptor 2 (HER2) negative, we limited our analysis to invasive tumors with this receptor subtype. Tumors that were estrogen receptor (ER) and/or progesterone receptor (PR) positive were considered HR-positive. Histology codes were used to identify cohorts, with the ILC cohort comprising those with codes for ILC or mixed ILC/IDC (histology codes 8520 and 8524 if behavior was invasive), and the IDC cohort comprising codes for IDC or invasive mammary carcinoma not otherwise specified (histology codes 8500, 8501, 8502, 8503, and 8523 if behavior was invasive). We excluded patients with stage I-III disease, those with breast cancer histologic subtypes other than IDC or ILC, individuals who died within 6 months of their diagnosis, and those who were missing critical clinical information including disease stage, HR status, HER2 status, or type of treatment received.

### Clinical Measures

Charlson-Deyo Co-Morbidity Index was recorded as a measure of severity of co-morbid conditions. Age at diagnosis was subdivided into under 50 years and over 50 years to estimate pre- and post-menopausal status, respectively. The sites of metastatic disease were categorized as bone-only, visceral-only, bone and visceral, or unknown [4]. We utilized available data on treatment facility type, insurance status, and median income in univariate and multivariate analyses.

### Statistical Methods

We compared clinicopathologic and demographic features between the ILC and IDC cohorts using chi-square tests for categorical variables and t-tests for continuous variables. We investigated factors associated with receiving surgery for the primary tumor, radiotherapy, chemotherapy, and timing of chemotherapy relative to surgery by histologic subtype. For univariate analyses, we used Kaplan-Meier plots and log-rank tests to assess the association between receipt of surgery and OS by histologic subtype, and by timing of chemotherapy and receipt of radiotherapy. We also evaluated treatment facility type (community versus academic), insurance type, and median income quartiles by surgery and histologic subtype.

For multivariate analysis, we used Cox proportional hazards models to account for confounders with OS. The multivariable model included age, Charlson-Deyo score (0/1+), site of metastasis (bone-only versus all other), and receptor subtype (ER-positive, PR-positive, HER2-negative versus ER-positive, PR-negative, HER2-negative).

Finally, we performed propensity score matching to account for likelihood of having primary site surgery to determine the association between primary site surgery and OS on multivariable analysis in matched ILC and IDC cohorts. The propensity score matched model included age, tumor grade, receptor subtype, site of metastatic disease, Charlson-Deyo score (0/1+), and treating facility variables. Within each histology category among those who had survival data available patients who had surgery (ILC n = 1,444; IDC n = 4,924) were matched to patients who did not have surgery (ILC n = 3,553; IDC n = 10,894) using the greedy nearest neighbor matching algorithm in which one control unit is matched with each unit in the treated group (1:1), producing the smallest within-pair difference among all available pairs. Matching was restricted to observations that had propensity scores in the extended common support region (ILC 0.05–0.71; IDC 0.06–0.66), which extends the common support region by 0.25 times a pooled estimate of the common standard deviation of the logit of the propensity score. The PSMATCH procedure in SAS version 9.4 was used to perform matching. Acceptable balance was defined by a maximum of 0.2 for the absolute value of standardized difference and by values within the 0.5-2 range for variance ratio. To account for the matched nature of the sample, log-rank tests and Cox models were stratified on the matched pairs.

Hypothesis tests were two-sided, and the significance threshold was set to 0.05. Statistical analyses were performed using Stata 16 and SAS version 9.4.

## Results

### Study Cohort

**Figure 1:**
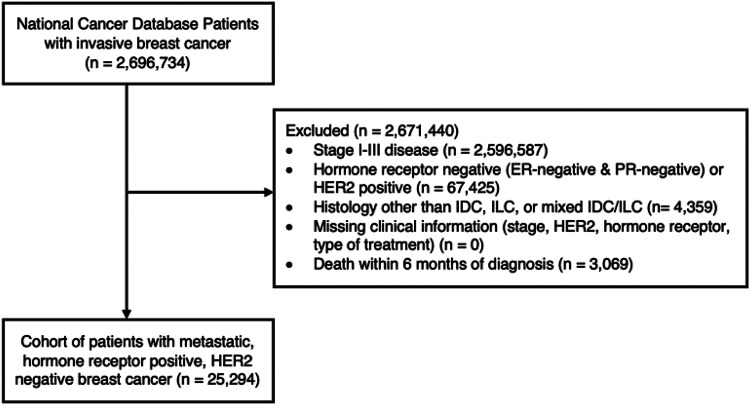
CONSORT flow diagram for study population of stage IV, hormone receptor positive, HER2-negative breast cancer patients from the National Cancer Database (PUF 2010 - 2016). *ER,* estrogen receptor; *PR,* progesterone receptor; *HER2,* human epidermal growth factor receptor 2; *IDC,* invasive ductal carcinoma; *ILC,* invasive lobular carcinoma.

There were 100,147 patients with stage IV breast cancer in the NCDB, of whom 25,294 had HR-positive, HER2-negative invasive lobular or ductal histology, and met the study criteria (Fig. 1). Of these patients, 19,171 (75.8%) had IDC and 6,123 (24.2%) had ILC. The median follow-up time of the ILC cohort was 27.2 months (interquartile range [IQ] 14.7–41.5 months), which was similar to the median follow-up time of 26.8 months (IQ range 14.6–42.6 months) for the IDC cohort (Table 1). Patients in the ILC cohort were slightly older than those in the IDC cohort (mean age 64 years versus 61 years, *p* < 0.001) and differed significantly by race (*p* < 0.001), with a higher proportion of White patients (79.4% versus 74.2%, *p* < 0.0001) (Table 1). Additionally, ILC and IDC patients differed significantly by tumor grade (*p* < 0.001), with ILC patients having a higher proportion of grade 1 tumors (22.1% versus 9.48%, p < 0.0001) and more often had bone-only metastases than those with IDC (60.8% versus 45.2%, *p* < 0.001).

While overall most patients were treated at community cancer centers, slightly more ILC patients than IDC patients were treated at academic centers (ILC 36.0% versus IDC 33.5%, *p* = 0.001) (Table 1). ILC patients were significantly more likely to have public insurance (ILC 54.7% versus IDC 51.6%) and less likely to be uninsured (ILC 3.82% versus IDC 5.15%) (*p* < 0.0001). Patients with ILC had higher median income, with 39.7% in the highest quartile compared to 35.7% of IDC patients in the highest median income quartile (*p* < 0.0001).

### Primary Site Surgery by Histologic Subtype

**Figure 2:**
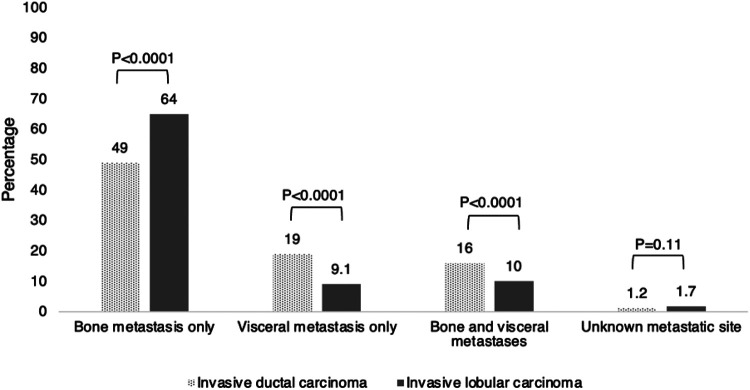
Distribution of metastatic sites by histologic subtype among patients who underwent primary site surgery.

In the entire study population of 25,294 patients with metastatic HR-positive HER2-negative breast cancer, 7,158 (28.3%) underwent primary site surgery. Although the absolute difference is small, primary site surgery was performed less often in those with metastatic ILC than those with metastatic IDC (n = 1,644 [26.8%] versus n = 5,514 [28.8%] respectively, *p* = 0.004) (Table 2). This remained true regardless of site of metastatic disease (bone-only, visceral-only, bone and visceral, and unknown site, Fig. 2). Among those who had primary site surgery in both the ILC and IDC cohorts, the site of metastatic disease was significantly more likely to be bone-only compared to other sites (ILC 63.5%; IDC 48.5%, *p* < 0.001) (Table 3, Fig. 2).

For both the ILC and IDC cohorts, patients with private insurance were significantly more likely to receive primary site surgery (ILC 46.4%; IDC 49.4%, *p* < 0.0001) compared to patients with public insurance (Table 3). Both ILC and IDC patients who received surgery were equally likely to have been treated at an academic treating facility (ILC n = 415 [26.3%]; IDC n = 1,318 [26.2%]) compared to a community setting.

Among the patients who underwent primary site surgery (n = 7,158), the differences between those with ILC and IDC reflected those seen in the overall study population. Those with ILC were older (ILC mean age 61.7 years versus IDC 58.1 years, *p* < 0.001), had larger tumors (ILC 31.6% T3 versus IDC 15.5% T3, *p* < 0.001), were more likely to have N3 nodal status (ILC 34.6% versus 18.2, *p* < 0.001), and were more likely to have grade 2 disease (ILC 61.4% versus 45.9%, *p* < 0.001). There was no significant difference in the type of surgery between the two cohorts with 1,166 (70.9%) patients with ILC having mastectomy and 3,788 (68.7%) patients with IDC having mastectomy (Table 2). Positive surgical margins were significantly more common in those with ILC compared to those with IDC (10.6% versus 8.3%, *p* = 0.005) (Table 1).

The factors associated with undergoing primary site surgery versus not having primary site surgery were similar in the ILC and IDC cohorts. In both groups, primary site surgery was less common in older patients, and was more common in those with larger tumors (except T4) and higher N category (Table 4). The odds of undergoing primary site surgery were highest for ILC patients with pathologic stage T3 disease versus T1 (OR 2.65, 95% CI 1.17–3.51, p = 0.002; Table 4) whereas the odds of surgery for patients with IDC were highest in pathologic stage T2 disease versus T1 (OR 1.71, 95% CI 1.30–2.25, p < 0.001). In both groups, those with T4 disease had significantly lower odds of primary site surgery compared to T1 (Table 4). Over time, the odds of undergoing primary site surgery decreased. In both the ILC and IDC cohorts, the odds of surgery decreased by 16% per each additional year of diagnosis (OR 0.84 and p < 0.001 for both groups; 95% CI 0.82–0.87 in ILC group; 95% CI 0.83–0.85 in IDC group).

### Radiotherapy by Histologic Subtype

The use of radiotherapy overall was lower in those with ILC than those with IDC (29.1% versus 37.9%, *p* < 0.001) (Table 2). In IDC patients who had surgery, 51.5% also had radiation, while in ILC patients who had surgery, 42.5% also had radiation (Table 3). Among those who received radiotherapy, there was no significant difference in the rate of radiation to local versus distant sites by histologic subtype (*p* = 0.55).

### Use and Timing of Chemotherapy

In these cohorts of patients with HR-positive HER2-negative metastatic disease, more patients with IDC received chemotherapy compared to patients with ILC (47.4% versus 41.3%, *p* < 0.001), while those with ILC were more likely to receive endocrine therapy (83.5% versus 78.6%, *p* < 0.001). For those who had primary-site surgery, the sequence of chemotherapy and surgery differed by histologic subtype; while 40.5% of patients with IDC had chemotherapy prior to surgery, only 29.0% of patients with ILC had chemotherapy prior to surgery (*p* < 0.001).

### Survival Analyses in Unmatched Cohorts

**Figure 3:**
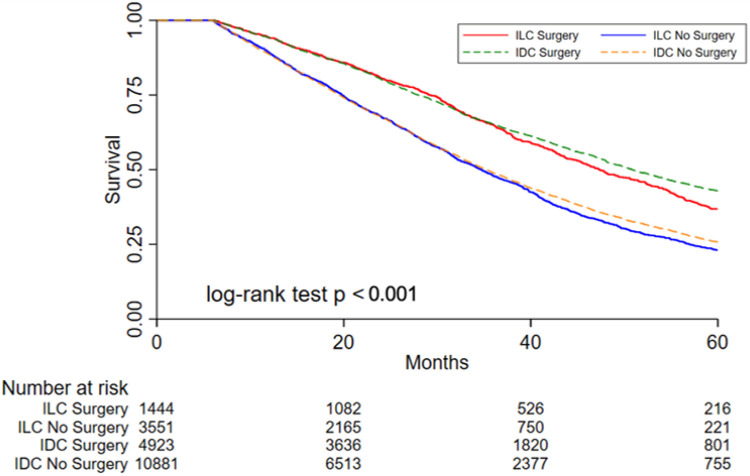
Kaplan-Meier survival curves based on primary site surgery and histology for stage IV hormone receptor positive, HER2-negative patients in the National Cancer Database (PUF 2010 - 2016). Estimated overall survival in patients with invasive ductal carcinoma (IDC) and invasive lobular carcinoma (ILC) with or without surgery (unmatched, unadjusted cohorts).

Overall, patients with metastatic ILC had slightly but significantly shorter OS than those with metastatic IDC (median 38 months versus 40 months respectively, *p* = 0.006). In both the ILC and IDC cohorts, having surgery for the primary tumor was associated with significantly improved OS (Table 1). In the ILC cohort, undergoing primary site surgery was associated with 35% lower risk of death compared to those who did not undergo surgery (HR 0.65, 95% CI 0.57–0.68, *p* < 0.001) (Fig. 3). This association persisted when controlling for age, Charlson-Deyo (1 + versus 0), site of metastases, and receptor subtype (HR 0.64, 95% CI 0.58–0.70, *p* < 0.001). The timing of surgery (before or after systemic chemotherapy) was not significantly associated with OS among those with ILC in unadjusted analysis nor after controlling for age, Charlson-Deyo (1 + versus 0), site of metastases, and receptor subtype.

Similarly, patients in the IDC cohort who underwent primary site surgery had an associated 40% lower risk of death compared to those without surgery (HR 0.60, 95% CI 0.57–0.63, *p* < 0.001) (Fig. 3). This remained true after controlling for age, Charlson-Deyo (1 + versus 0), site of metastasis, and receptor subtype (HR 0.61, 95% CI 0.58–0.64, *p* < 0.001). Unlike for those with ILC, the timing of surgery was significantly associated with OS. Patients with IDC who had chemotherapy before surgery had 24% less risk of death compared to those who had surgery prior to chemotherapy (HR 0.76, 95% CI 0.70–0.84, *p* < 0.001). This association persisted after controlling for age, Charlson-Deyo (1 + versus 0), site of metastasis, and receptor subtype (HR 0.83, 95% CI 0.76–0.92, *p* < 0.001).

The type of surgery (mastectomy versus lumpectomy) was not significantly associated with different OS in either ILC or IDC groups. However, we did find a significant statistical interaction between having surgery for the primary tumor and the site of radiation (local versus distant). Primary site surgery was associated with a greater reduction in risk of death among those who had local radiation compared to those who had distant radiation (HR 0.35, 95% CI 0.3–0.4 versus HR 0.67, 95% CI 0.61–0.74 respectively, test of interaction *p* < 0.001). This interaction between primary site surgery and site of radiation was similar in both the ILC and IDC cohorts separately.

### Survival Analyses in Propensity Score Matched Cohorts

The propensity score model included age, tumor grade, receptor subtype, site of metastatic disease, Charlson-Deyo score (0/1+), and treating facility variables, with 3089 ILC patients (991 with surgery, 2,098 without surgery) and 11,216 IDC patients (3,429 with surgery, 7,787 without surgery) in each cohort with available data for matching. In both the ILC and IDC matched samples there was still a significant association between having surgery for the primary tumor and improved OS (HR 0.71, 95% CI 0.59–0.85, *p* < 0.001 for ILC cohort; HR 0.67, 95% CI 0.61–0.74, *p* < 0.001 for IDC cohort).

## Discussion

Recent randomized trial data suggest that the role of primary site surgery in the management of patients with metastatic breast cancer is limited to local control in select cases, with no clear evidence of impact on overall survival rates [9, 10, 11]. However, how to best select these patients who might garner some benefit from primary site surgery remains unknown, with these decisions being made on an individualized basis in clinical practice [13]. Given the known differences between lobular and ductal tumors in regard to surgical management in the early stage setting and disease patterns in the metastatic setting, we explored whether the use of primary site surgery differs in HR-positive HER2-negative metastatic lobular versus ductal breast cancer.

In this cohort of 25,294 patients from the National Cancer Database, we found both similarities and differences in the management of metastatic HR-positive HER2-negative breast cancer by histologic subtype. First, despite the lack of definitive data demonstrating a survival benefit from primary site surgery, a significant proportion of patients overall underwent primary site surgery (28.3%). While primary site surgery was more commonly utilized in those with bone-only metastases, and those with ILC were more likely to have such a disease pattern, the overall usage of primary site surgery in the ILC cohort was slightly but significantly lower than in the IDC cohort. Although a slightly smaller proportion of patients with ILC had primary site surgery, the majority of factors associated with receiving surgery did not differ between the lobular and ductal groups; in both groups, primary site surgery was more common among younger patients, those with T2 or T3 tumors, those with more nodal disease, and those with private insurance.

Interestingly, while patients with ILC had larger tumors than those with IDC, there was no difference in the rate of mastectomy by histologic subtype amongst those who had primary site surgery. This differs from the early-stage setting, where lobular histology is associated with higher mastectomy rates. Similar to the early-stage setting, however, those with metastatic ILC who had primary site surgery experienced significantly higher positive margin rates than those with metastatic IDC. This raises the possibility that the local control benefit of primary site surgery might be attenuated in those with ILC, who may require more extensive surgery to achieve negative margins. We did find an association between local radiotherapy and improved overall survival in this cohort; whether this association reflects a relationship between improved local control and survival outcomes versus improved outcomes in those selected to have radiation is unknown. Of note, patients with ILC were significantly less likely to receive radiation than those with IDC, which is consistent with other studies [16, 17].

One interesting finding regarding management of the primary tumor in this metastatic cohort is the significantly lower odds of primary site surgery in patients with T4 tumors in both lobular and ductal groups. Since the most accepted purpose of primary site surgery in the stage IV setting is for palliation, we would have expected higher rates of surgery in those with T4 tumors. Alternatively, these tumors may have been deemed unresectable; one of the challenges of analyzing this retrospective dataset is the inability to discern the reasons for performing primary site surgery.

This limitation likely impacts the strong association between primary site surgery and improved OS that we found in both ILC and IDC patients. For example, we found that in both the ILC and IDC cohorts, patients who had private insurance were more likely to have surgery compared to patients who had public insurance. The improved outcomes associated with primary site surgery may reflect improved access to care as opposed to a biologic effect of surgery. While we attempted to adjust for potential confounders in the propensity score matched model, we are likely unable to account for the many factors that influence why surgery would be used in some patients versus others.

Of more interest, perhaps, is the finding that the use of pre-operative systemic therapy was associated with improved OS in the IDC cohort, but not in the ILC cohort. We suspect that pre-operative systemic therapy in the IDC cohort may have helped to select patients who would have more durable response to therapy, and therefore have improved OS. In contrast, response to therapy in those with ILC may be more difficult to ascertain, or less likely to be associated with outcomes.

For systemic therapy, those with ILC were significantly more likely to receive endocrine therapy than those with IDC, despite all studied cases being HR-positive. Likewise, those with IDC were more likely to receive chemotherapy. This treatment pattern has been observed in previous literature and may point to the notion that early-stage ILC has reduced sensitivity to chemotherapies, or perceived as such, and therefore utilized less frequently [18, 19]. However, more recent studies show that in the metastatic setting, response to eribulin and CDK4/6 inhibitors may be similar between ILC and IDC [20, 21]. These findings highlight the need to identify lobular specific therapies for those with metastatic disease.

As a secondary endpoint, we also looked at OS by histology. Similar to our findings, worse OS in those with metastatic ILC has been shown in other studies as well [2, 3, 22]. While the underlying reason for this difference is unclear, it suggests that ILC is indeed biologically different than IDC, given differential outcomes despite restricting the study population to those with HR-positive, HER2-negative tumor types, and ILC tumors being of lower grade than IDC tumors. One potential explanation could be that those with metastatic ILC may have an overall higher burden of disease than is typically detected on standard imaging modalities [23]. For example, studies comparing fluorodeoxyglucose positron emission tomography (FDG-PET) to ^18^F-fluoroestradiol positron emission tomography (FES-PET) show that some metastatic lesions in ILC appear only on FES-PET [24, 25].

To our knowledge, this is the largest reported study evaluating primary site surgery by histologic subtype in the setting of metastatic breast cancer. However, this study is subject to a number of limitations, including selection bias, lack of detailed systemic therapy information, lack of specific radiation field data, and the absence of local recurrence events as an endpoint. However, the findings reflect real-world management patterns which appear to differ by histologic subtype.

While ILC has long been regarded as a less aggressive tumor type, our findings from this large NCDB study are consistent with others showing worse outcomes in ILC than IDC. While the differences between the IDC and ILC groups in this study were relatively small, it is interestingly to note that histology appears to be influencing management among this HR-positive HER2-negative group of patients with stage IV breast cancer. The use of primary site surgery was slightly lower, and the use of both radiotherapy and chemotherapy were much lower in those with metastatic ILC compared to metastatic IDC. Coupled with shorter overall survival in the ILC cohort, these findings reinforce the need for histologic subtype-specific management options. In regard to surgical management, the significantly larger tumor size and higher positive margin rates in the ILC cohort suggest that if primary site surgery is to be utilized, one should consider a larger excision and likely incorporate radiotherapy to maximize potential benefit of locoregional intervention. Whether such outcomes reflect under-staging, and the need to better identify disease extent in ILC, or lack of ILC specific treatments is unknown. Regardless, further work is needed to improve management outcomes for those with metastatic ILC.

## Figures and Tables

**Table 1:** Comparison of patient characteristics between invasive ductal carcinoma (IDC) and invasive lobular carcinoma (ILC) cohorts in unmatched population.

Patient Characteristics	IDC, n (%) Total n = 19,171	ILC, n (%) Total n = 6,123	*P*-value
**Mean Age** [S.D.]	61.3 [± 13.9]	63.6 [± 12.6]	<0.0001
Age < 50	3,861 (20.1%)	836 (13.7%)	<0.0001
Age ≥ 50	15,310 (79.8%)	5,287 (86.3%)	
**Median Follow up time**, months [IQ range]	26.8 [14.6–42.6]	27.2 [17.7–41.5]	
**Race**			
White	14,116 (74.2%)	4,825 (79.4%)	<0.0001
African American	3,010 (15.8%)	753 (12.4%)	
East Asian	130 (0.683%)	27 (0.444%)	
Other	1,763 (9.27%)	470 (7.74%)	
**Hispanic**			0.19
Hispanic	1,081 (5.82%)	319 (5.36%)	
Non-Hispanic	17,508 (94.2%)	5,629 (94.6%)	
**Treatment Facility Type**			0.001
Academic	6,003 (33.5%)	2146 (36%)	
Community^a^	11,927 (66.5%)	3810 (64%)	
**Primary Payer**			<0.0001
No insurance	987 (5.15%)	234 (3.82%)	
Private insurance	7,990 (41.7%)	2,475 (40.4%)	
Public insurance	9,890 (51.6%)	3,348 (54.7%)	
Unknown insurance	304 (1.59%)	66 (1.08%)	
**Median Income Quartiles** ^b^			<0.0001
<$40,227	3,724 (19.7%)	1,030 (17.1%)	
$40,227–50,353	4,053 (21.4%)	1,212 (20.1%)	
$50,354–63,332	4,389 (23.2%)	1,398 (23.2%)	
≥$63,333	6,739 (35.6%)	2,394 (39.7%)	
**Receptor Status**			0.017
ER positive / PR positive	16,170 (84.3%)	5,086 (83.1%)	
ER positive / PR negative	3,001 (15.7%)	1,037 (16.9%)	
**Grade**			<0.0001
1	1,580 (9.48%)	1,020 (22.1%)	
2	8,822 (52.9%)	2,861 (62%)	
3	6,272 (37.6%)	732 (15.9%)	
**Charlson-Deyo Score**			0.007
0	15,894 (82.9%)	4,984 (81.4%)	
≥1	3,277 (17.1%)	1,139 (18.6%)	
**Positive Surgical Margins**	444 (8.33%)	169 (10.6%)	0.005
**Metastasis site**			<0.0001
Bone only	8,510 (45.2%)	3,641 (60.8%)	
All other	10,334 (54.8%)	2,346 (39.2%)	
**Overall Survival in months (Median (95% CI))**			
All patients	39.7 (39.0-40.6)	38.4 (47.2–39.7)	0.006
With surgery	50.9 (49.1–52.9)	47.4 (44.9–50.6)	<0.001
Without surgery	35.3 (34.4–36.0)	34.7 (33.5–35.9)	<0.001

a Community treatment facility includes Community Cancer Programs, Comprehensive Community Cancer Programs, and Integrated Network Cancer Programs

b Median Income Quartiles from 2012–2016

**Table 2:** Comparison of treatment patterns by histology. *IDC*, invasive ductal carcinoma; *ILC*, invasive lobular carcinoma; *CNS*, central nervous system.

Treatment	IDC, n (%) Total n = 19,171	ILC, n (%) Total n = 6,123	*p*-value
**Any surgery**	5,514 (28.8%)	1,644 (26.8%)	0.004
**Surgery type**			0.19
Lumpectomy	1,621 (27.5%)	445 (27.1%)	
Mastectomy	3,788 (64.2%)	1,166 (70.9%)	
Radical mastectomy	105 (8.38%)	33 (2.01%)	
**Chemotherapy**			<0.001
No chemotherapy	9,592 (50%)	3,406 (55.6%)	
Yes chemotherapy	9,084 (47.4%)	2,529 (41.3%)	
Unknown chemotherapy	495 (2.58%)	188 (3.1%)	
**Chemotherapy timing**			<0.001
Preoperative chemotherapy	2,231 (40.5%)	477 (29%)	
Postoperative chemotherapy	3,283 (59.5%)	1,167 (71%)	
**Endocrine therapy**			<0.001
No endocrine therapy	3,520 (18.4%)	869 (14.2%)	
Yes endocrine therapy	15,075 (78.6%)	5,112 (83.5%)	
Unknown endocrine therapy	576 (3%)	142 (2.32%)	
**Any radiation therapy**	7,260 (37.9%)	1,781 (29.1%)	<0.001
**Radiation location**			0.55
Local radiation	2,365 (35.9%)	564 (35.1%)	
Distant radiation	4,220 (64.1%)	1,042 (64.9%)	
**Detailed radiation location**			
CNS/head	600 (8.26%)	168 (9.43%)	0.02
Viscera	86 (1.18%)	28 (1.57%)	
Breast	2,365 (3.25%)	564 (31.7%)	
Bone	3,534 (48.7%)	846 (47.5%)	
Other	383 (5.28%)	79 (4.43%)	
Unknown	292 (4.02%)	96 (5.4%)	

**Table 3:** Sociodemographic and treatment patterns broken down by histology and surgery status. Total number of patients, (n= ), unless otherwise stated. *IDC*, invasive ductal carcinoma; *ILC*, invasive lobular carcinoma; *CNS*, central nervous system.

	Invasive Ductal Carcinoma				Invasive Lobular Carcinoma			
	All IDC (n = 19,171)	IDC w/surgery (n = 5,514)	IDC w/out surgery (n = 13,657)	*P*-value	All ILC (n = 6,123)	ILC w/surgery (n = 1,644)	ILC w/out surgery (n = 4,479)	*P*-value
**Treatment Facility** ^a^				<0.0001				<0.0001
Academic	6,003 (33.5%)	1,318 (26.2%)	4,685 (36.3%)		2,146 (36%)	415 (26.3%)	1,731 (39.6%)	
Community	11,927 (66.5%)	3,714 (73.8%)	8,213 (63.7%)		3,810 (64%)	1,164 (73.7%)	2,646 (60.4%)	
**Primary Payer** ^b^								
No insurance	987 (5.15%)	207 (3.75%)	780 (5.71%)	<0.0001	234 (3.82%)	41 (2.49%)	193 (4.31%)	<0.0001
Private insurance	7,990 (41.7%)	2,723 (49.4%)	5,267 (38.6%)		2,475 (40.4%)	763 (46.4%)	1,712 (38.2%)	
Public insurance	9,890 (51.6%)	2,509 (45.5%)	7,381 (54.1%)		3,348 (54.7%)	824 (50.1%)	2,524 (56.4%)	
Unknown	304 (1.59%)	75 (1.36%)	229 (1.68%)		66 (1.08%)	16 (1.12%)	50 (1.12%)	
**Median Income Quartile** ^c^				<0.0001				<0.0001
<$40,227	3,724 (19.7%)	1,084 (20.0%)	2,640 (19.6%)		1,030 (17.1%)	260 (16.1%)	770 (17.4%)	
$40,227–50,353	4,053 (21.4%)	1,175 (21.6%)	2,878 (21.4%)		1,212 (20.1%)	351 (21.7%)	861 (19.5%)	
$50,354–63,332	4,389 (23.2%)	1,261 (23.2%)	3,128 (23.2%)		1,398 (23.2%)	365 (22.6%)	1,033 (23.4%)	
≥$63,333	6,739 (35.7%)	1,911 (35.2%)	4,828 (35.8%)		2,394 (39.7%)	641 (39.6%)	1,753 (39.7%)	
**Metastatic Site**								
Bone metastasis only	8510 (44.4%)	2,677 (48.5%)	5833 (42.7%)	<0.001	3641 (59.5%)	1044 (63.5%)	2597 (58%)	<0.001
Visceral metastasis only	3226 (16.8%)	1,059 (19.2%)	2167 (15.9%)	<0.001	515 (8.4%)	150 (9.12%)	365 (8.15%)	<0.001
Bone and visceral metastases	5424 (28.3%)	873 (15.8%)	4551 (33.3%)	<0.001	1167 (19.0%)	163 (9.91%)	1004 (22.4%)	<0.001
Unknown metastatic site	2011 (10.5%)	905 (16.4%)	1106 (8.1%)	<0.001	800 (13.1%)	287 (17.5%)	513 (11.5%)	<0.001
**Radiation frequency**	7260 (37.9%)	2,842 (51.5%)	4418 (32.3%)	<0.001	1781 (29.1%)	698 (42.5%)	1083 (24.2%)	<0.001
**Radiation binary**				<0.001				<0.001
Local	2,365 (35.9%)	1835 (69.2%)	530 (13.5%)		564 (35.1%)	458 (69.5%)	106 (11.2%)	
Distant	4,220 (64.1%)	817 (30.8%)	3,403 (86.5%)		1042 (64.8%)	201 (30.5%)	841 (88.8%)	
**Radiation detailed**				<0.001				<0.001
CNS/head	600 (8.26%)	87 (3.06%)	513 (11.6%)		168 (9.43%)	18 (2.58%)	150 (13.9%)	
Viscera	86 (1.18%)	39 (1.37%)	47 (1.06%)		28 (1.57%)	8 (1.15%)	20 (1.84%)	
Breast	2365 (32.6%)	1,835 (64.6%)	530 (12%)		564 (31.7%)	458 (65.6%)	106 (9.79%)	
Bone	3534 (48.7%)	691 (24.3%)	2843 (64.4%)		846 (47.5%)	175 (25.1%)	671 (62%)	
Other	383 (5.28%)	100 (3.52%)	283 (6.41%)	79 (4.44%)	18 (2.58%)	61 (5.63%)
Unknown	292 (4.02%)	90 (3.17%)	202 (4.57%)	96 (5.39%)	21 (3.01%)	75 (6.93%)

a Treatment facility data available for 17,930 IDC and 5,956 ILC patients

b Primary payer data available for 19,171 IDC and 6,123 ILC patients

c Median income quartile data available for 18,905 IDC and 6,034 ILC patients

**Table 4:** Factors associated with receiving primary site surgery in those with metastatic invasive ductal carcinoma and those with metastatic invasive lobular carcinoma. *OR odds ratio; CI confidence interval*.

	Unadjusted OR	95% CI	*p*-value
**Invasive Ductal Carcinoma**			
Age^a^	0.83	(0.81–0.85)	<0.001
Stage 0 vs T1	0.50	(0.31–0.81)	0.0045
Stage T2 vs T1	1.71	(1.30–2.25)	0.0001
Stage T3 vs T1	1.68	(1.17–2.41)	0.0050
Stage T4 vs T1	0.63	(0.49–0.82)	0.0006
Node N1 vs N0	0.73	(0.60–0.88)	0.0008
Node N2 vs N0	4.21	(3.18–5.58)	<0.001
Node N3 vs N0	2.91	(2.17–3.91)	<0.001
Year of diagnosis	0.84	(0.83–0.85)	<0.001
**Invasive Lobular Carcinoma**			
Age^a^	0.85	(0.81–0.89)	<0.001
Stage 0 vs T1	0.08	(0.04–0.20)	<0.001
Stage T2 vs T1	2.03	(1.17–3.51)	0.0113
Stage T3 vs T1	2.65	(1.43–4.90)	0.0020
Stage T4 vs T1	0.45	(0.27–0.78)	0.0042
Node N1 vs N0	0.79	(0.57–1.09)	0.1529
Node N2 vs N0	19.65	(9.28–41.61)	<0.001
Node N3 vs N0	12.12	(6.88–21.35)	<0.001
Year of diagnosis	0.84	(0.82–0.87)	<0.001

a In this analysis, patient age at diagnosis is scaled to every 10 years.

## Data Availability

The datasets analyzed during the current study are available in the National Cancer Database, https://www.facs.org/quality-programs/cancer-programs/national-cancer-database/.

## Abbreviations

ILC: Invasive lobular carcinoma
IDC: Invasive ductal carcinoma
OS: Overall survival
NCDB: National Cancer Database
HR: Hormone receptor
HER2: Human epidermal growth factor receptor 2
ER: Estrogen receptor
PR: Progesterone receptor
FDG-PET: fluorodeoxyglucose positron emission tomography
FES-PET: ^18^F-fluoroestradiol positron emission tomography
